# Reduced endothelial dependent vasodilation in vessels from TLR4^−/−^ mice is associated with increased superoxide generation

**DOI:** 10.1016/j.bbrc.2011.04.024

**Published:** 2011-05-20

**Authors:** Louise S. Harrington, Martina H. Lundberg, Michael Waight, Adrian Rozario, Jane A. Mitchell

**Affiliations:** Cardiothoracic Pharmacology, NHLI, Imperial College, Dovehouse Street, London SW3 6LY, UK

**Keywords:** TLR, Toll like receptor, DHE, dihydroethidium, DAPI, 4′,6-diamidino-2-phenylinodole, Nox, NADPH oxidase, U46619, 9,11-dideoxy-11α,9α-epoxymethanoprostaglandin F_2α_, PSS, physiological saline solution, Ach, acetylcholine, SNP, sodium nitroprusside, SOD, superoxide dismutase, Toll like receptor 4, Basal nitric oxide, Superoxide, Vasodilation, Artery, Dihydroethidium

## Abstract

Toll like receptor (TLR)4 is a pattern recognition receptor expressed in endothelial and other cells, responsible for the sensing of endotoxin and host derived ligands. Our group has shown previously that the absence of TLR4 is associated with reduced endothelial dependent vasodilator responses and left heart hypertrophy in animal models. However, the mechanism behind reduced endothelial cell function in TLR4^−/−^ mice is not known.

We have used *en face* confocal imaging of mesenteric arteries from mice deficient in the TLR4 receptor stained with dihydroethidium (DHE) to measure superoxide production. Using the isometric wire myograph, mesenteric artery vasodilator responses to acetylcholine and MnCl_2_ (a superoxide dismutase mimetic) were measured. Mesenteric arteries from TLR4^−/−^ mice had a reduced endothelial dependent relaxant response and increased superoxide levels when stimulated with acetylcholine. Increased levels of superoxide, as detected by DHE staining, were seen in vessels from TLR4^−/−^ mice, which were reduced to control levels in the presence of MnCl_2_.

Our observations suggest that loss of TLR4 increases superoxide generation which reduces the biological activity of endothelial derived nitric oxide and thereby explains the endothelial dysfunction and associated cardiovascular phenotype in TLR4^−/−^ mice. These data implicate a novel cardio-protective role for TLR4 in vascular homeostasis.

## Highlights

► The absence of TLR4 is associated with reduced endothelial cell function in mesenteric arteries. ► TLR4^−/−^ mouse arteries have reduced acetylcholine responses. ► TLR4^−/−^ have increased superoxide levels when stimulated with acetylcholine. ► An increase in superoxide generation reduces the activity of endothelial derived nitric oxide.

## 1 Introduction

Endothelial cells mediate vasodilation by the release of vasoactive hormones including nitric oxide and prostacyclin [1]. In most blood vessels, including those used in this study, nitric oxide is the dominant endothelial vasodilator hormone [2]. The biological activity of nitric oxide is critically influenced by levels of superoxide anions and the quenching of superoxide radicals results in enhanced vasodilation in bioassay systems when endothelial cells are stimulated [3]. We have recently shown that endothelial dependent vasodilation is significantly reduced in vessels from mice lacking the pattern recognition receptor, Toll like receptor (TLR)4 [4]. Others have shown that TLR4^−/−^ mice express increased levels of superoxide in isolated lung endothelial cells which leads to emphysema as the mice age [5]. In the current study we have tested the hypothesis that blood vessels from TLR4^−/−^ mice have increased superoxide levels and assessed how this may affect endothelial function in blood vessels from these mice [6].

## 2 Materials and methods

Female homozygous TLR4^−/−^ mice on a C57 Black 6 background [7] were provided by Sandra Sacré, Michael Goddard and Claudia Monaco (Kennedy Institute, London, UK). Age matched inbred wild type C57 Black 6 mice were obtained from Charles River (UK) and Harlan (UK). The mice were maintained and killed humanely in accordance with the EC Directive 86/609/EEC for animal experiments.

Genotype was confirmed by PCR of DNA extracted from ear clips using Qiagen spin columns. PCR amplification was carried out using GE Healthcare PuRe PCR beads and the following primers: forward primer: cgt gta aac cag cca ggt ttt gaa ggc, reverse primer: tgt tgc cct tca gtc aca gag act ctg, and neomycin resistance gene primer: atc gcc ttc tat cgc ctt ctt gac gag. The reaction was cycled 35 times with 94 °C (for 15 s) denaturing, 60 °C (for 15 s) annealing and 72 °C (for 1 min) elongation temperatures.

For isometric wire myography, vessel segments from wild type mice aged 10–16 weeks (12.9 ± 0.7 weeks) and TLR4^−/−^ mice aged 11–16 weeks (13.0 ± 0.7 weeks) were used. Mice were killed by cervical dislocation and mesenteric arteries removed and prepared as described previously [4]. Arteries were dissected free of fat and connective tissues, and the vessel segments placed in Mulvany wire myographs [8] to measure contraction and relaxation responses. Throughout the experiment, tissues were immersed in physiological salt solution (PSS) 1.18 × 10^−1^ mol/L NaCl, 4.7 × 10^−3^ mol/L KCl, 1.17 × 10^−3^ mol/L MgSO_4_, 2.5 × 10^−3^ mol/L CaCl_2_, 1.0 × 10^−3^ mol/L KH_2_PO_4_, 2.7 × 10^−5^ mol/L EDTA, and 5.5 × 10^−3^ mol/L glucose, at 36 °C, bubbled with 95% O_2_ and 5% CO_2_. The tension of the vessel was normalised to a tension equivalent to that generated at 90% of the diameter of the vessel at 100 mmHg. Changes in arterial tone were recorded via a PowerLab/800 recording unit (ADI instruments Pty Ltd., Australia), and analysed using Chart 6.0 acquisition system (ADI instruments).

Arteries were exposed to two challenges of high potassium solution (KPSS; 1.24 × 10^−1^ mol/L KCl, 1.17 × 10^−3^ mol/L MgSO_4_, 2.5 × 10^−3^ mol/L CaCl_2_, 1.0 × 10^−3^ mol/L KH_2_PO_4_, 2.7 × 10^−5^ mol/L EDTA, and 5.5 × 10^−3^ mol/L glucose) followed by a washout period of 10 min. Concentration response curves to 9,11-dideoxy-11α,9α-epoxymethanoprostaglandin F_2α_ (U46619; 10^−9^–3 × 10^−7^ mol/L) were performed on each of the tissues. Dilatory response curves were recorded in arteries pre-contracted with an EC_80_ concentration of U46619, and vasodilator responses to either acetylcholine (10^−8^–10^−4^ mol/L) or MnCl_2_ (10^−8^–3 × 10^−4^ mol/L) were assessed.

To image and quantify superoxide levels, wild type mice aged 9–11 weeks (mean 10.3 ± 0.2 weeks) and TLR4^−/−^ mice aged 8–11 weeks (mean 9.8 ± 0.8 weeks) were used. Some arteries were exposed to the superoxide dismutase mimetic MnCl_2_ (3 × 10^−4^ mol/L) for 20 min prior to contracting the mesenteric arteries with EC_80_ 9,11-dideoxy-11α,9α-epoxymethanoprostaglandin F_2α_ (U46619). Arteries were left contracted for 10 min before the addition of dihydroethidium (DHE; 10^−5^ mol/L), and a further 20 min before the addition of acetylcholine at increasing concentrations [4]. DHE binds with superoxide to form 2-hydroxyethidium which can be imaged using confocal microscopy. At the end of these experiments tissues were fixed with 2% paraformaldehyde for 15 min. 4′,6-Diamidino-2-phenylinodole (DAPI; 7 × 10^−6^ mol/L) was added for 5 min to visualise the nuclei. Arteries were mounted for confocal microscopy using hard-set vectashield medium, and images captured the following day using a Leica SP5 inverted microscope with LAS AF (2.0.2) software.

Detection wavelength settings were 406–458 nm for DAPI (blue), and 575–700 nm for DHE (red) imaging. The gain and offset for each channel were determined in pilot studies, using a wild type Ach control artery to indicate maximum staining (set just below the saturation point). Following optimisation, all microscope settings were rigorously adhered to.

Images through the *Z* plane of the whole mesenteric artery were achieved by locating the endothelial layer by morphology of the DAPI stained nuclei (rectangular, running perpendicular to the smooth muscle cells’ nuclei), and an image of the red DHE stained endothelial cells was taken. The focus was then changed to the smooth muscle cells above the endothelial cells indicated by the blue DAPI stained nuclei (elongated nuclei wrapping around the artery), and an image then taken of the red DHE staining. Due to differences in shape and size of each artery, the total pixel count was taken from within a rectangle of 168 μm × 183 μm moved within each image to exclude non-artery image. From each tissue, three sets of images (endothelial cells and the overlying smooth muscle cells) along the length of the artery were taken from each individual mouse.

## 3 Results

**Fig. 1:**
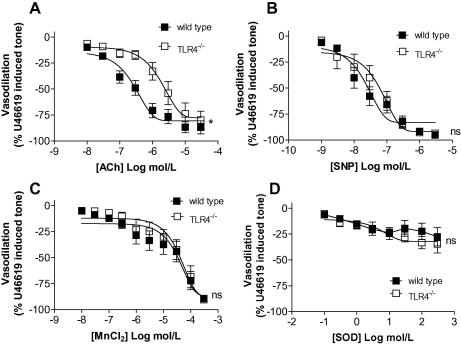
Dilatory responses in arteries from wild type and TLR4^−/−^ mice, pre-contracted with EC_80_ U46619. Dilation induced by (A) acetylcholine (Ach); *n* = 11 for wild type and *n* = 10 for TLR4^−/−^ vessels. (B) SNP; *n* = 11 for wild type and *n* = 10 for TLR4^−/−^ vessels. (C) MnCl_2_; *n* = 10 for wild type and *n* = 5 for TLR4^−/−^ vessels. (D) Superoxide dismutase (SOD); *n* = 8 for wild type and *n* = 5 for TLR4^−/−^ vessels. Data are represented as mean ± SEM. ^∗^*p* < 0.05; ns, not significant by two-way ANOVA.

**Fig. 2:**
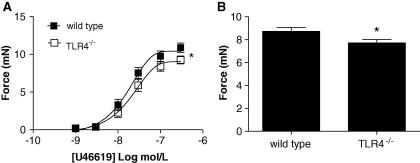
Contractile responses in arteries from wild type and TLR4^−/−^ mice to (A) U46619; *n* = 27 for wild type and *n* = 20 for TLR4^−/−^ vessels. Data are represented as mean ± SEM. ^∗^*p* < 0.05 by two-way ANOVA; and (B) high [K^+^] *n* = 26 for wild type and *n* = 20 for TLR4^−/−^ vessels. Data are represented as mean ± SEM. ^∗^*p* < 0.05 by unpaired *t*-test.

Each mouse genotype was confirmed to being a homozygous wild type or TLR4^−/−^ by PCR. As we have shown previously [4], vessels from TLR4^−/−^ mice had reduced endothelial dependent vasodilator response when stimulated with acetylcholine (Fig. 1A). Endothelium independent vasodilation induced by sodium nitroprusside (SNP) was unaffected by the deletion of the TLR4 gene (Fig. 1B). Vasodilation induced by the cell permeable superoxide dismutase mimetic, MnCl_2_ [9], was similar in tissues from wild type and TLR4^−/−^ mice (Fig. 1C). Addition of authentic superoxide dismutase (SOD), which does not enter cells readily, did not induce vasodilation in arteries from either wild type or TLR4^−/−^ mice (Fig. 1D). Contractile responses to U46619 (Fig. 2A) and high [K^+^] (Fig. 2B) were modestly reduced in arteries from TLR4^−/−^ mice compared with those from wild type controls.

**Fig. 3:**
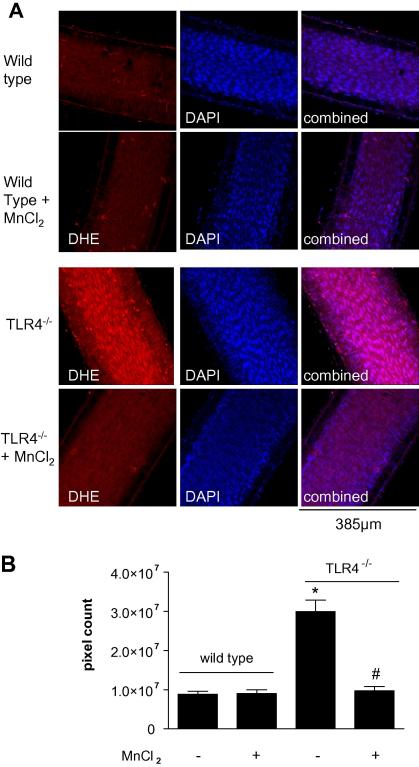
Superoxide levels in the endothelial cell layer of (A) wild type and TLR4^−/−^ arteries, with or without MnCl_2_. Left hand side images show arteries incubated with DHE which produces 2-hydroxyethidium when oxidised by ${\text{O}}_{2}^{-}$; middle images show blue fluorescence of the nuclear stain DAPI; right hand side images show DHE and DAPI combined. (B) Data are represented as mean pixel count ± SEM. ∗*p* < 0.05 compared to wild type; ^#^*p* < 0.05 for plus MnCl_2_ versus TLR4^−/−^ control by one-way ANOVA followed by Bonferroni’s Multiple Comparison Test, *n* = 6 mice. (For interpretation of the references to colour in this figure legend, the reader is referred to the web version of this article.)

**Fig. 4:**
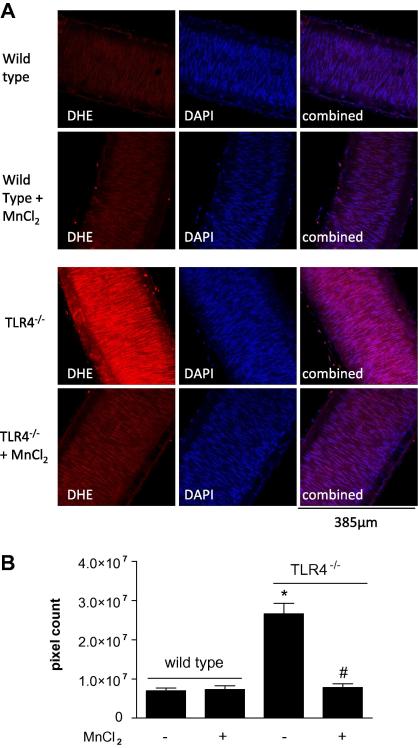
Superoxide levels in the smooth muscle cell layer of (A) wild type and TLR4^−/−^ arteries, with or without MnCl_2_. Left hand side images show arteries incubated with DHE which produces 2-hydroxyethidium when oxidised by ${\text{O}}_{2}^{-}$; middle images show blue fluorescence of the nuclear stain DAPI; right hand side images show DHE and DAPI combined. (B) Data are represented as mean pixel count ± SEM. ∗*p* < 0.05 compared to wild type; ^#^*p* < 0.05 for plus MnCl_2_ versus TLR4^−/−^ control by one-way ANOVA followed by Bonferroni’s Multiple Comparison Test, *n* = 6 mice. (For interpretation of the references to colour in this figure legend, the reader is referred to the web version of this article.)

In parallel experiments superoxide levels in the endothelial cell (Fig. 3) and smooth muscle cell (Fig. 4) layers of mesenteric arteries were visualised and quantified in vessels from wild type and TLR4^−/−^ mice. In vessels from wild type mice, superoxide levels were relatively low and not significantly inhibited by MnCl_2_. By contrast levels of superoxide were greatly increased in both endothelial cell and smooth muscle cell layers in arteries from TLR4^−/−^ mice and reduced to control levels in the presence of MnCl_2_ (Figs. 3 and 4).

## 4 Discussion

The ability of endothelial cells to release nitric oxide is an essential part of normal vascular homeostasis. Nitric oxide from the endothelium limits smooth cell proliferation, vasoconstriction and platelet reactivity by activating soluble guanylate cyclase leading to increased cGMP [1]. Nitric oxide is very unstable with a half life of few seconds in aqueous solution [3]. However, the biological half life of nitric oxide is greatly increased when superoxide anions are removed using dismutase enzymes or molecules [3]. Consequently, where superoxide anions are elevated the biological activity of nitric oxide is greatly reduced [10].

In previous studies from our group we have shown that nitric oxide mediated endothelial dependent vasodilator responses in murine vessels are greatly reduced in mice lacking TLR4 receptors [4]. Others have shown that lung microvascular endothelial cells from TLR4^−/−^ mice have increased expression of the respiration, and superoxide forming enzyme NADPH oxidase (Nox)3 [5]. If the same phenomenon occurs in endothelial cells lining arteries, increased superoxide generation could explain the reduced endothelial-dependent vasodilator effect [10].

We show using confocal imaging that vessels from TLR4^−/−^ mice have greatly increased levels of superoxide in both endothelial cell and smooth muscle cell layers. DHE binds superoxide to form 2-hydroxyethidium, or with hydrogen peroxide to form ethidium [11]. Our observations were validated and found to be specific for superoxide, since the superoxide dismutase mimetic MnCl_2_ greatly reduces fluorescence intensity. Moreover, endothelial dependent vasodilation induced by MnCl_2_ [9], unlike that induced by acetylcholine, was not limited in vessels from TLR4^−/−^ mice. In this study, as in others [12] authentic SOD did not induce meaningful dilator responses, this is likely due to the fact that the molecule is large and does not access cells and tissues easily. Acetylcholine activates the endothelium, increasing intracellular calcium which stimulates nitric oxide synthase to release nitric oxide [1]. Activation of the endothelium by acetylcholine would also activate Nox enzymes and increase superoxide production. By contrast MnCl_2_ induces endothelium-dependent vasodilation, without activating the endothelium, by removing superoxide and thereby enhancing the activity of ‘basally released’ nitric oxide. Whilst not conclusive, our observations showing differential effects of acetylcholine and MnCl_2_ in vessels from TLR4^−/−^ arteries are, therefore, consistent with our conclusions that increased superoxide generation in blood vessels accounts for dysfunction in endothelium-dependent vasodilation seen in vessels from TLR4^−/−^ mice. Interestingly we found that vessels from TLR4^−/−^ mice had modestly, but significantly, reduced contractile responses to U46619 or depolarising concentrations of K^+^. This could be a result of overall vascular dysfunction associated with increased superoxide production in the tissue. Indeed we observed increased superoxide levels in both endothelial cell and smooth muscle cell layers of the arteries.

These findings highlight a homeostatic role for TLR4 in cardiovascular function and explain why vessels from TLR4^−/−^ mice have compromised endothelial responses at the level of nitric oxide. Activation of TLR4 is generally considered to lead to pro-inflammatory effects in blood vessels and is linked to the development of septic shock [13] and atherosclerosis [14]. The human TLR4 polymorphisms Asp299Gly and Thr399Ile impair receptor function, and have been linked to a strong increase in acute coronary syndrome [15]. Yet in different reports, the Asp299Gly polymorphism was associated with a reduced risk of atherosclerosis [16], while another found a significant reduction in acute coronary events associated with Asp299Gly [17] compared to controls. These contrasting observations could be explained by the inevitable balance between the loss of TLR4 function in immune versus non-immune vascular cells. Loss of TLR4 on immune cells will lead to immune suppression and increased susceptibility to infection but on the other hand protection of non-immune host tissue from inflammation is associated with TLR4 activation. However, our data clearly illustrate a novel role for TLR4 in limiting superoxide generation and offering cardio-protection. We do not yet know if this is a direct effect of TLR4 or secondary to changes in the levels of infection/inflammation.

## Sources of funding

This work was funded by the Wellcome Trust.

## Conflict of interest disclosure

None.
